# Synthesis and Comparative Biological Properties of Ag-PEG Nanoparticles with Tunable Morphologies from Janus to Multi-Core Shell Structure

**DOI:** 10.3390/ma11101787

**Published:** 2018-09-20

**Authors:** Mengda Xu, Jie Liu, Xiankui Xu, Shanhu Liu, František Peterka, Yanrong Ren, Xianfeng Zhu

**Affiliations:** 1 Henan Engineering Laboratory of Flame-retardant and Functional Materials, Institute of Functional Polymer Composites, College of Chemistry and Chemical Engineering, Henan University, Kaifeng 475004, China; 104753150813@vip.henu.edu.cn (M.X.); 104753160807@vip.henu.edu.cn (J.L.); 104753170806@vip.henu.edu.cn (X.X.); liushanhu@henu.edu.cn (S.L.); 2 Technical University of Liberec, Studentská 1402/2 461 17, Liberec 46117, Czech Republic; fpet@volny.cz; 3 School of Life Sciences, Henan University, Kaifeng 475004, China

**Keywords:** silver nanoparticle, PEG, Janus nanoparticle, muli-core shell nanoparticle, antibacterial activity, cytotoxicity

## Abstract

Silver nanoparticles synthesized with polymers as coating agents is an effective method to overcome their poor stability and aggregation in solution. Silver-polyethylene glycol (Ag-PEG) nanoparticles were synthesized with the thiol-functionalized polyethylene glycol (SH-PEA) as the coating, reducing and stabilizing agent. The UV irradiation time, polymer and silver nitrate concentration for the synthesis were investigated. The concentration of silver nitrate had significant effect on the morphology of Ag-PEG nanoparticles. When increasing the concentration of silver nitrate, SEM and TEM images showed that Ag-PEG nanoparticles changed from Janus to multi-core shell structure. Meanwhile, pure silver particles in the two hybrid nanoparticles presented spherical shape and had the similar size of 15 nm. The antibacterial activities and cytotoxicity of the two structural Ag-PEG nanoparticles were investigated to understand colloid morphology effect on the properties of AgNPs. The results of antibacterial activities showed that the two structural Ag-PEG nanoparticles exhibited strong antibacterial activities against *Staphylococcus aureus*, *Escherichia coli* and *Bacillus subtilis*. The Janus nanoparticles had larger minimal inhibitory concentration (MIC) and minimum bacterial concentration (MBC) values than the multi-core shell counterparts. The results of cytotoxicity showed the Janus Ag-PEG nanoparticles had lower toxicity than the multi-core shell nanoparticles.

## 1. Introduction

Silver nanoparticles (AgNPs) have attracted increasing interest due to their unique physical, chemical and biological properties, including surface-enhanced Raman scattering, catalytic activity, non-linear optical behavior and broad-spectrum bactericidal activity [1,2,3,4,5,6,7,8,9]. Many methods have been reported for the synthesis of AgNPs with chemical, physical, photochemical and biological routes with tunable size and shape [10,11,12,13,14,15]. However, AgNPs have high surface area, resulting in poor colloidal stability and aggregation in solution. Stabilizing/capping agents have been utilized in the synthesis process of AgNPs to overcome this instability [16,17,18]. Among these agents, polymers are effective agents to form an outer shell coating to the silver cores or a carrier to loading AgNPs on their surface, providing a steric barrier to aggregation [19,20,21,22]. Furthermore, studies have been found that the concentration or the types of polymers have some effect on the size, shape and properties of AgNPs [11,18], while the effect of the morphology of Ag-polymer hybrids on their properties has rarely been concerned.

Janus particles possess two or more different chemical components and spatial distribution that have different surface, structures or material properties [23,24]. Although Janus nanoparticles combine individual components together, their properties are not interfered with or completely lost, which makes them a unique category of materials compared to conventional nanoparticles [25,26]. In comparison with the other noble metals, silver has gained less interest in the fabrication of Janus particles. Some silver-gold bimetal or silver-sillica Janus nanoparticles have been prepare by depositing growth on the metal seeds [27,28,29,30]. Chen et al. [27] had compared a Janus Au@Ag nanoparitlces to their Au@Ag and Ag@Au core-shell counterparts, and found that the Janus nanoparticles exhibited enhanced electrocatalytic activity in oxygen reduction reactions. They explained the result may be caused by the partial charge transfer from Au to Ag that optimized the oxygen absorption on the metal surfaces which was affected by the interfaces between Au and Ag. This research has illustrated that the properties of metal nanoparticles can be improved by varying their morphology. Up to date, few silver-polymer Janus nanoparticles have been studied in spite of the wide synthesis and application of silver-polymer hybrids.

In this paper, we present a facile and fast approach for the fabrication of silver-polyethylene glycol (Ag-PEG) nanoparticles with tunable morphologies in Janus and multi-core shell structure which was performed by photochemical reduction method with thiol-functionalized polyethylene glycol (SH-PEA) as the coating, reducing and stabilizing agent. Optimization of different experimental variables like UV irradiation time, polymer concentration and silver precursor concentration were systematically investigated for the two structures of Ag-PEG nanoparticles. Silver colloids are usually stable against auto-aggregation which is potential materials in antibacterial and anticancer medicine. Thus, the biological properties of Ag-PEG nanoparticles had been concerned about. To understand the effect of the morphology of the hybrid nanoparticles on their biological properties, the antibacterial activities and cytotoxicity of the Janus and multi-core shell Ag-PEG nanoparticles were assessed by bacterial growth inhibitory and 3-[4,5-dimethylthiazol-2-yl]-2,5-dephenyl tetrazolium bromide (MTT) cell viability assays, respectively.

## 2. Materials and Methods 

### 2.1. Materials

Mercapto-ethylamine (MEA) was purchased from Xiya Reagent (Linshu, Shandong, China). Poly(ethylene glycol) diglycidyl ether (PEGDE, *M*_n_ = 500 g·mol^−1^) was obtained from Sigma-Aldrich Co. (Shanghai, China). Silver nitrate (AgNO_3_), alcohol and n-hexane were purchased from Tian Jin Chemical Reagent (Tianjin, China). 3-(4,5-dimethylthiazol-2-yl)-2,5-ziphenyl tetrazolium bromide (MTT) was purchased from Amresco Inc. (Solon, OH, USA). Dulbecco’s modified eagle medium (DMEM) and fetal bovine serum (FBS) were obtained from Gibco BRL Co. (Gaithersburg, MD, USA). The phosphate buffer saline (PBS) was made in laboratory according to the literature [31]. Briefly, potassium chloride (4 g), potassium phosphate monobasic (4 g), sodium chloride (160 g), and sodium phosphate dibasic heptahydrate (43.2 g) were dissolved in 1 L deionized (DI) water. *Staphylococcus aureus* (ATCC 6358P), *Escherichia coli* (CGMCC 1.90) and *Bacillus subtilis* (CGMCC 1.1849) were used for the bacterial studies. All microorganisms were provided by China General Microbiological Culture Collection Center (CGMCC), Chinese Academy of Sciences (CAS), Beijing, China. The bacterial strains were grown in Luria-Bertani agar plates from Hopebiol (Qingdao, Shandong, China) at 37 °C.

### 2.2. Measurement

FT-IR spectra were recorded in a FT-IR SPECRUM VERTEX 70 spectrometer (Bruker, Ettlingen, Germany) on thoroughly dried samples by using KBr pellets. ^1^H Nuclear Magnetic Resonance (^1^H NMR) spectra were collected on an AVANCE III HD 400 MHz spectrometer (Bruker, Zurich, Switzerland) in CDCl_3_ at 25 °C. Gel permeation chromatography (GPC) measurements were conducted with a Waters 410 GPC (Waters, Milford, PA, USA) equipped with Waters Styragel column (HT4 + HT3) using CDCl_3_ as the eluent, the molecular weights were calibrated with polystyrene standards, and the flow rate was set at 1.0 mL min^−^^1^ at 35 °C. UV-Vis absorption spectra were recorded by using a U4100 spectrophotometer (Hitachi, Shanghai, China) with the as-prepared silver colloidal solution. The surface morphology of samples was analyzed on a JSM-7610F (JEOL, Tokyo, Japan) scanning electron microscope (SEM) and the main elements were measured by energy-dispersive X-ray spectrometry (EDS) (JEOL, Tokyo, Japan). The samples on the silicon wafers were mounted rigidly to a copper specimen holder using a conductive adhesive. Transmission electron microscopy (TEM) studies were performed on a JEM-2100 electron microscope (JOEL, Tokyo, Japan) operating at an acceleration voltage of 100 KV. The nanoparticle solution was dropped on copper grids, and dried at room temperature. X-ray diffraction (XRD) was recorded on a D 8 Advance (Bruker, Karlsruhe, Germany). The samples were drop cast on glass slides, and dried at room temperature.

### 2.3. Synthesis of Thiol-Functionalized Polyethylene Glycol (SH-PEA)

**Scheme 1:**
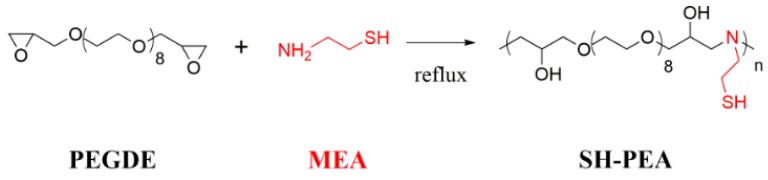
Schematic illustration of SH-PEG synthesis.

SH-PEA was synthesized following the procedure conducted by Wen and coworkers [31]. A mixture of PEGDE (0.001 mol) and mercapto-ethylamine (0.001 mol) was dissolved by ethanol (4 mL) with nitrogen (Air Separation Group Co., Ltd., Kaifeng, Henan, China). The mixture was stirred for 12 h under refluxing. Then, the mixture was poured into n-hexane. After removing the supernatant, the product was collected and dried in vacuum oven (Bluepard Instrumets Co., Ltd., Shanghai, China) at 40 °C. The synthesis process was described in Scheme 1.

### 2.4. Synthesis of Ag-PEG Nanoparticles

The synthesis of Ag-PEG nanoparticles was carried out in a typical experiment. SH-PEA (20 mg) and AgNO_3_ (4 mg) were dissolved into 20 mL distilled water at room temperature. After 3 h, the mixture solution was subjected to UV-irradiation with a UV light (UV LED Curing System, UP313, Uvata Precision Optoelectronics Co., Ltd., Shanghai, China) at an intensity of 75 mW·cm^−2^ at 365 nm for 3 min. The reaction mixture was extensively dialyzed against water for 3 days to remove the excess reactants. The optimization of different experimental variables of UV irradiation time, SH-PEA concentration and silver nitrate concentration were investigated systematically.

### 2.5. Antibacterial Properties of Ag-PEG Nanoparticles

Oxford cup method was used to determine the antibacterial activity with different bacterial such as *E. coli*, *S. aureus* and *B. subtilis* according to the previous report [32]. The bacterial suspension (10^6^ CFU mL^−1^) was inoculated evenly on a Luria-Bertani agar plate. Then, the Oxford cups were placed on the surfaces of the cultures, and an amount of 200 μL solution of the nanoparticle (1 mg mL^−1^) was place into the oxford cups. After incubation for 24 h at 37 °C, the antibacterial activity was evaluated by measuring the average diameters of the zones of inhibition with a vernier calliper for three times.

The minimal inhibitory concentration (MIC) was read by the visual turbidity of the tubes noted before and after incubation according to previous reports [33,34]. The Ag-PEG nanoparticles were dissolved in a nutrent agar to prepare stock solutions (4 mg mL^−1^). Distinct volumes of the stock solutions were diluted by 10 mL growth medium to form series of solutions with different concentration of Ag-PEG nanoparticles. Then, these solutions were inoculated with 100 μL of a bacterial suspension (10^5^ CFU mL^−1^) and then cultured for 1 day in a 37 °C incubator and immediately used in MIC assays. To determine MIC, the cultures were analyzed for the absence of turbidity. A total of 3 replicates were carried out for each treatment and the corresponding controls.

The minimum bacterial concentration (MBC) was determined according to the previous report [35]. After the MIC determination of the Ag-PEG nanoparticles tested, an aliquot of 1 mL from each test tube in which no visible bacterial growth was observed was inoculated on Luria-Bertani agar and then incubated overnight at 37 °C. MBC is defined as the lowest concentration of antimicrobial agent that kill >99% of the initial bacterial population, at which there is the lowest concentration of antimicrobial agent that on the plates have no bacterial growth. Each bacterium was assayed at least three times.

### 2.6. Cytotoxicity

The cytotoxicity of Ag-PEG was examined by MTT assay. All sample solutions were diluted with DMEM to obtain preset concentrations. HepG2 and MCF-7 cells were seeded into 96-well plates with a density of 10^4^ cells per well and incubated in DMEM (100 mL) for 24 h. Then, seven concentrations (250, 125, 64, 32, 6, 8 and 4 μg mL^−1^) of Ag-PEG were added to the wells. Three parallel wells for each sample were used at a specific concentration. After co-incubation with cells for 48 h, 20 μL of MTT solution in PBS (5 mg mL^-1^) was added to each well and the plate was incubated for another 4 h at 37 °C. After that, the medium containing MTT was removed, and 150 μL of DMSO was added to each well to dissolve the MTT formazan crystals. Finally, the plates were shaken for 5 min, and the absorbance of formazan product was measured at 490 nm by a Bio-Rad 680 micro-plate reader (Hercules, CA, USA).

## 3. Results and Discussion

### 3.1. Characterization of the SH-PEA

PEG is a hydrophilic polymer with excellent biocompatibility and widely used to modify metal or metal oxide nanomaterials for biomedicine application [36,37,38], which makes PEG the appropriate polymer for fabrication of AgNPs for the application in biological systems. The thiol-functionalized PEG was synthesized through nucleophilic-addition/ring-opening reaction between the epoxy groups of PEGDE and the amine group of MEA (Scheme 1). Nitrogen atoms were introduced into the macromolecular backbone after the reaction. The nitrogen-containing polymers have been successfully utilized as reducing and stabilizing agents in one-pot synthesis of AgNPs directly in aqueous solution without the aid of any other external agent [39,40]. Thus, it is interesting to use SH-PEA as the reducing and stabilizing agent for the fabrication of AgNPs due to the nitrogen atoms assisting silver ions reduction and the coordination of thiol groups to the surface as-formed AgNPs.

**Figure 1:**
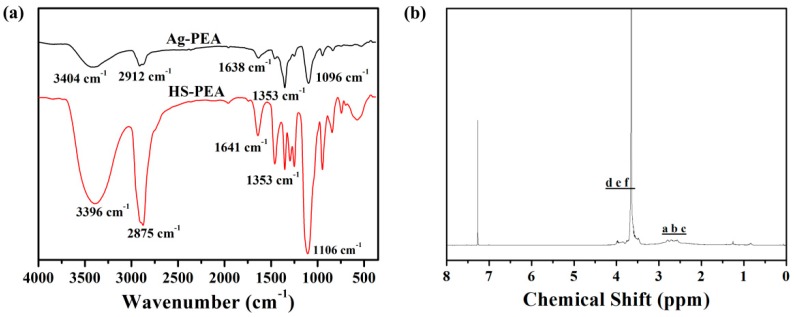
(**a**) FT-IR spectra of Ag-PEG nanoparticles and SH-PEA and (**b**) ^1^H NMR spectrum of SH-PEA.

FT-IR and ^1^H NMR spectra were used to study chemical structure of SH-PEA. SH-PEA has one characteristic absorption peak at 3396 cm^−1^, attributed to the stretching vibration of the -OH groups. The peaks at 2875 cm^−1^ belongs to the stretching vibration of C-H bond (Figure 1a). The asymmetric stretching vibration of C-O-C of SH-PEA appears at 1106 cm^−1^. ^1^H NMR spectrum of SH-PEA is shown in Figure 1b. The signals in the range of 2.4–2.8 ppm are assigned to -CH_2_- adjoining the *ter*-amino groups. The methylene and methine protons of the main chains give the signals at 3.4–4.1 ppm. Both FT-IR and ^1^H NMR data confirm the structure of SH-PEA as proposed. GPC was utilized to determine the molecular weights *M*_n_ and polydispersity (*M*_w_*/M*_n_). The data shows the *M*_n_ and polydispersity for SH-PEA is 0.67 × 10^4^ g mol^−1^ and 2.41.

### 3.2. Synthesis of Ag-PEG Nanoparticles

The functionality of SH-PEA makes it possible to utilizing photochemical reduction route for the fabrication of AgNPs with a green and facile method. Photochemical reduction has been proved as a cost-effective, convenient and controllable technique to prepare AgNPs [41,42,43]. In this work, Ag-PEG nanoparticles were prepared through photochemical reduction of AgNO_3_ with SH-PEA in water.

The formation of Ag-PEG nanoparticles was confirmed by FT-IR spectroscopy. As shown in Figure 1a, the spectra of the Ag-PEG nanoparticles exhibit a few differences from that of SH-PEA. The peaks at 1641 cm^−1^, corresponding to the stretching peaks of tert-amine, shifts to 1638 cm^−1^ with a significant decrease in the transmittance. Meanwhile, the intensity of the C-S stretching band around 656 cm^−1^ almost disappears in the spectrum of Ag-PEG nanoparticles.

**Figure 2:**
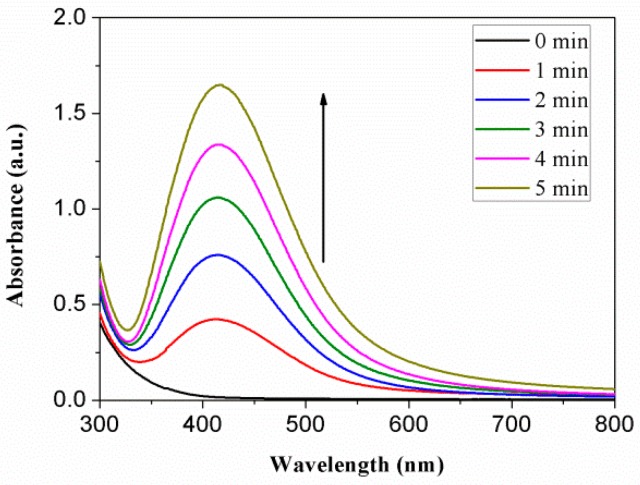
UV-Vis absorption spectra of Ag-PEG nanoparticles with different irradiation time.

The optical properties of Ag-PEG nanoparticles were investigated using UV-Vis spectrometry. Figure 2 shows the UV-Vis absorption spectra of photochemical reduced products of a mixture of 0.2 mg mL^−1^ AgNO_3_ and 1 mg mL^−1^ SH-PEA at different time. It is obvious that no AgNPs formed without UV irradiation, even though the aqueous solution of SH-PEA and AgNO_3_ was stirred in dark for 3 h. However, the color of the solution changed from colorless to brown when the solution was irradiated for several minutes, indicating the reduction of silver ion to silver and the formation of AgNPs. An absorption band was existed at 414 nm after irradiation for 1 min, and the absorbance increases with increasing irradiation time. A single symmetric band with one maximum around 400 nm for the surface Plasmon resonance are attributed to the typical absorption spectrum of spherical AgNPs [44]. Although the nanoparticles irradiated for 5 min had the highest absorbance, the aggregation of the nanoparticles is serious and visible precipitation gathered at the bottom of the colloid solution. Thus, the irradiation time of 3 min is accepted for the other experiments for stable Ag-PEG nanoparticles.

**Figure 3:**
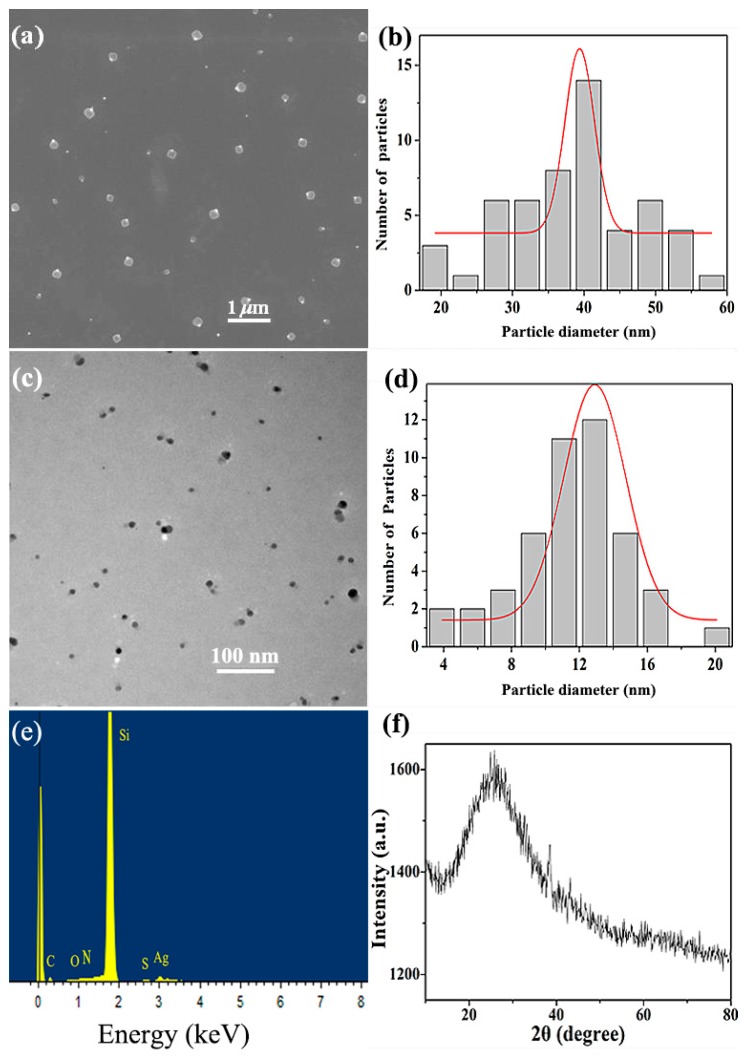
(**a**) SEM and (**c**) TEM images, (**b**,**d**) particles size distribution histograms from SEM and TEM images, (**e**) EDS image and (**f**) XRD pattern of Ag-PEG nanoparticles synthesized with 0.2 mg mL^−1^ AgNO_3_, 1 mg mL^−1^ SH-PEA and 3 min irradiation.

The morphologies and corresponding silver domain size distributions of Ag-PEG nanoparticles observed by using SEM and TEM are shown in Figure 3. The spherical AgNPs were ensured by SEM and TEM analysis. SEM image (Figure 3a) encloses a Janus structure with uniform dispersing which has about 38 ± 18 nm bright silver due to a strong electron scattering signal and around 100 nm darkened PEG domain. TEM image (Figure 3c) is a little different from SEM image, which shows the Janus nanoparticles have black spherical silver domains with the size about 13 ± 7 nm and grey PEG domains with the size about 15 nm. The difference size of PEG domains in SEM and TEM images may caused by the quickly drying process of TEM sample by using the filter paper remove the excess water of the sample. Both of the images illustrate the asymmetric nanoparticles disperse uniformly in water without agglomeration. EDS image explains the nanoparticles contain Ag, C, N, O and S elements, matching the components of as-synthesized Ag-PEG nanoparticles (Figure 3e). The XRD pattern of Ag-PEG nanoparticles is shown in Figure 3f. The broad peak with 2θ around 25.5° is related to the diffraction of amorphous SH-PEA, while the AgNPs show weak peaks at 2*θ* = 38.4° and 43.2° index to (111) and (200) planes of Ag.

**Figure 4:**
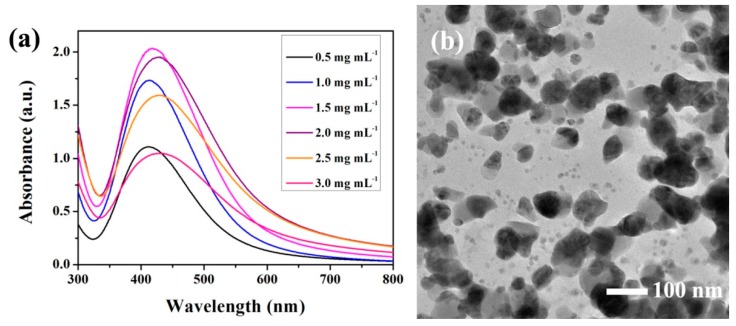
(**a**) Absorption spectra of Ag-PEG nanoparticles with different SH-PEA concentration; (**b**) TEM image of Ag-PEG nanoparticles synthesized with 2.5 mg mL^−1^ SH-PEA and 0.2 mg mL^−1^ AgNO_3_.

To investigate the influence of SH-PEA concentration on the size of AgNPs, we synthesized Ag-PEG nanoparticles with different SH-PEA concentration and 0.2 mg mL^−1^ AgNO_3_, and monitored their surface Plasmon resonance with a UV-Vis spectrometer. In Figure 4a, the surface Plasmon resonance shows a band with a maximum at 414 nm for the concentration of SH-PEA increasing from 0.5 to 1.5 mg mL^−1^. Furthermore, increasing SH-PEA concentration, the band turns broader and takes a red shift to 428 nm, which indicated that the AgNPs grow up or aggregate. Figure 4b shows the TEM image of Ag-PEG nanoparticles synthesized with 2.5 mg mL^−1^ SH-PEA. Large Ag-PEG nanoparticles and agglomeration are observed, and the size of pure AgNPs is over 50 nm, much larger than the size of Ag-PEG nanoparticles synthesized with 0.2 mg mL^−1^ SH-PEA. Besides, both Ag and PEG domains become irregular although the nanoparticles remain Janus structure. It is obvious that the lower SH-PEA concentration is appropriate for the stable Ag-PEG nanoparticles.

**Figure 5:**
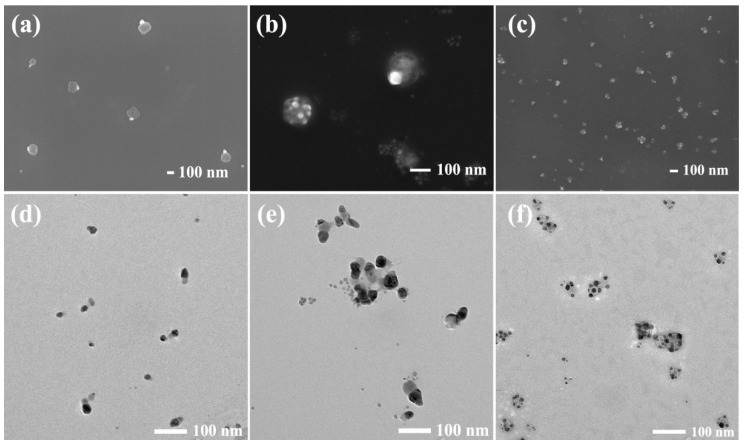
SEM images of Ag-PEG nanoparticles with (**a**) 0.3, (**b**) 0.5 and (**c**) 1.0 mg mL^−1^ AgNO_3_ concentration; TEM images of Ag-PEG nanoparticles with (**d**) 0.3, (**e**) 0.5 and (**f**) 1.0 mg mL^−1^ AgNO_3_ concentration.

AgNO_3_ used as the precursor is an important variable in the synthesis of AgNPs. However, few researches have studied the effect of silver ion on the size, shape or properties of AgNPs. In this synthesis process, we found that the concentration of AgNO_3_ played a key role in the morphology of Ag-PEG nanoparticles. Figure 5 shows the morphological transformation with varying AgNO_3_ concentration from 0.2 to 1 mg mL^−1^. Figure 5a,d are the SEM and TEM images of the nanoparticles synthesized with 0.3 mg mL^−1^ AgNO_3_, respectively. They are very similar as the result of Ag-PEG nanoparticles with 0.2 mg mL^−1^ AgNO_3_ shown in Figure 3, except for the larger Ag domains with the size about 25 nm. As AgNO_3_ concentration is 0.5 mg mL^−1^, a multi-core shell structure nanoparticles with the size about 100 nm exists in the image as shown in Figure 5b. It corresponding TEM image (Figure 5e) enclosed the multi-core shell structure may caused by the agglomeration of the Janus nanoparticles. Figure 5b,e both present the multi-core shell structure coexists with the Janus structure, and the size of Ag domain further increases to 40 nm. As AgNO_3_ concentration is up to 1.0 mg mL^−1^, the Janus nanoparticles almost disappear, and they are replaced by the multi-core shell nanoparticles (Figure 5c,f). The size of the multi-core shell nanoparticles is around 60 nm, and the size of Ag cores returns to 15 nm. According to the above results and discussion, it is concluded that the lower AgNO_3_ concentration is acceptable for Janus Ag-PEG nanoparticles and the higher AgNO_3_ concentration is appropriate for multi-core shell nanoparticles.

**Figure 6:**
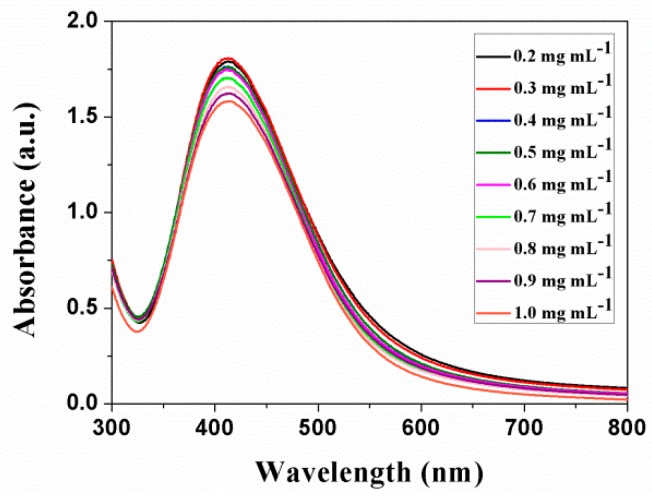
UV-Vis absorption spectra of Ag-PEG nanoparticles with different AgNO_3_ concentration.

Although the significant change had taken place in the morphology of Ag-PEG nanoparticles as varying AgNO_3_ concentration, their UV-Vis absorption spectra still present a single band with a maximum around 414 nm (Figure 6). Since the single band is relative to the surface Plasmon resonance of spherical AgNPs, the shape of Ag domains ought to keep spherical shape as increasing AgNO_3_ concentration from 0.2 to 1.0 mg mL^−1^. The UV-Vis spectra, SEM and TEM image all explain the shape of the pure AgNPs remains unchanged when the morphology of Ag-PEA transforms.

### 3.3. Antibacterial Activity

AgNPs not only have broad spectrum antibactericidal and fungicidal activity [1,2], but also show high toxicity to animal species and cultured cells [45]. Studies have found that the biological effect of AgNPs depend on their size, shape and surface coating [46,47]. In this work, we were more interested in the morphological effect of Ag-PEG nanoparticles on their biological effect. For the biological properties test, the Janus nanoparticles were synthesized with 0.2 mg mL^−1^ AgNO_3_ and 1 mg mL^−1^ SH-PEA, and the multi-core shell nanoparticles were with 1 mg mL^−1^ AgNO_3_ and 1 mg mL^−1^ SH-PEA.

**Figure 7:**
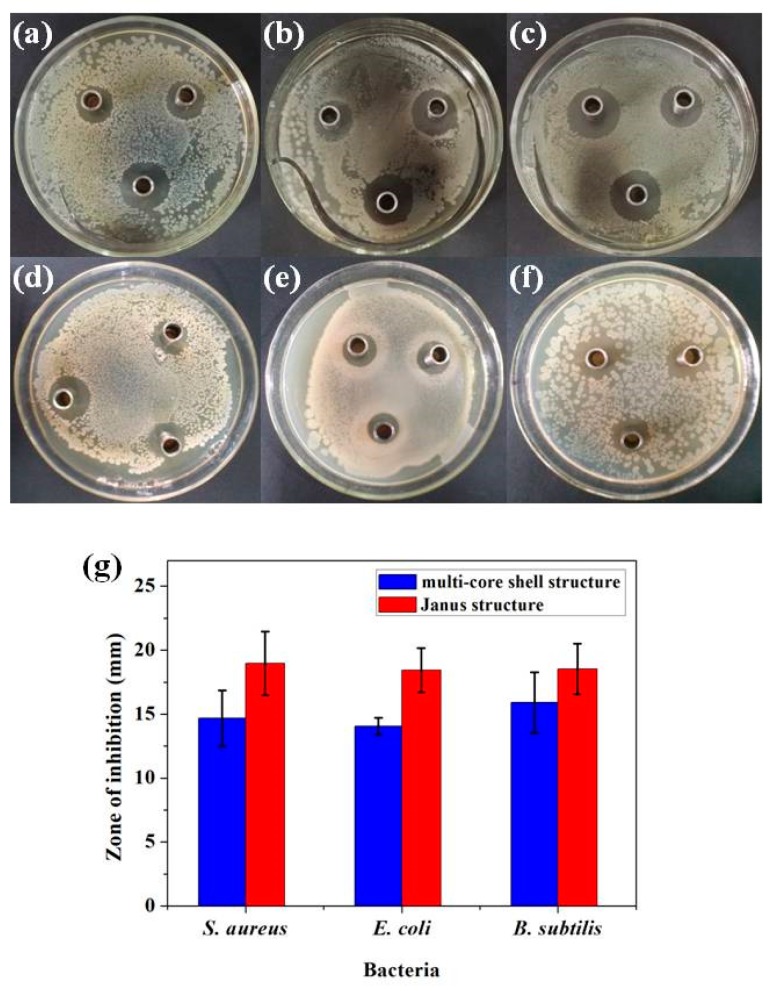
Inhibition Zones of the Janus (**a**–**c**) and multi-core shell Ag-PEG nanoparticles (**d**–**f**) against *S. aureus* (**a**,**d**), *E. coli* (**b**,**e**) and *B. subtilis* (**c**,**f**); (**g**) the diameter of the inhibition zone of Ag-PEG nanoparticles.

The antibacterial activity of SH-PEA and the Ag-PEG nanoparticles was evaluated by using bacterial growth inhibitory assay. Three bacteria were used to test the capability of different morphologies of Ag-PEG nanoparticles. One is Gram positive bacterium of *S. aureus*, the others are Gram negative bacteria of *E. coli* and *B. subtilis*. After incubated for 24 h at 37 °C, bacteria were still around the Oxford cups, which indicated SH-PEA did not have any antibacterial activity against the three bacteria. In Figure 7a–f, areas of clear media surround the Oxford cups filled with the solution of Janus or multi-core shell Ag-PEG nanoparticles. The average diameter of the zones of inhibition of the multi-core shell Ag-PEG nanoparticles are 14.67 ± 2.19, 14.05 ± 0.65 and 15.90 ± 2.37 mm against *E. coli*, *S. aureus* and *B. subtilis*, respectively as shown in Figure 7h. And the values of the Janus Ag-PEG nanoparticles are 18.96 ± 2.50, 18.42 ± 1.73 and 18.52 ± 1.99 mm, respectively. The results demonstrate the two structure Ag-PEG nanoparticles have strong antibacterial activities to inhibit the growth of the three bacteria, and each structural nanoparticles perform even activities. Moreover, the Janus Ag-PEG nanoparticles perform better antibacterial activity than the multi-core shell ones. It should be noted that the pure silver amount of the Janus samples is much less than that of the multi-core shell ones. Since the shape and size of the pure AgNPs are same, the result must be caused by the difference between their morphologies.

A large decrease in the optical absorbance of the bacterial suspensions was observed when they were exposed to Ag-PEG nanoparticles. As shown in Table 1, the minimal inhibitory concentration (MIC) values of Janus Ag-PEG nanoparticles were 64, 32 and 64 μg mL^−1^ against *S. aureus*, *E. coli* and *B. subtilis*, respectively. The values of multi-core shell nanoparticles were all 16 μg mL^−1^ against the three bacteria. The minimum bacterial concentration (MBC) values of the Janus nanoparticles were much higher than that of the multi-core shell nanoparticles. The MBC values of Janus Ag-PEG nanoparticles were 500 μg mL^−1^ against *S. aureus* and *E. coli*, and 250 μg mL^−1^ against *B. subtilis*, while the values of multi-core shell nanoparticles were 64 μg mL^−1^ against *S. aureus* and 32 μg mL^−1^ against *E. coli* and *B. subtilis*. The MBC/MIC ratio shows the two structural nanoparticles have different antibacterial properties. The ratio of the Janus Ag-PEG nanoparticles is higher than 4, suggesting that the Janus Ag-PEG can be considered as a bacteriostatic agent. On the contrary, the multi-core shell Ag-PEG nanoparticles have lower MBC/MIC ratio (≤4), performing with bactericidal manner [33].

### 3.4. Cytotoxicity

**Figure 8:**
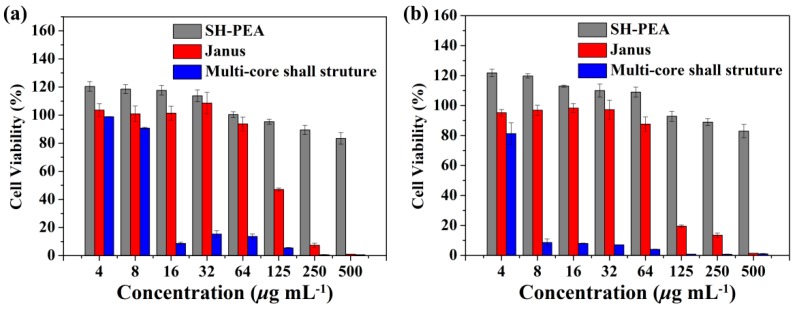
The cytotoxicity of SH-PEA and Ag-PEG nanoparticles against (**a**) HepG2 and (**b**) MCF-7 cells at different levels of concentration after 48 h incubation. Data were presented as mean ± standard deviation (*n* = 3).

MTT assay was used to evaluate the cytotoxicity of Ag-PEG nanoparticles by using HepG2 and MCF-7 cell lines, and the results were shown in Figure 8. After incubating in all cell lines for 48 h, the data clearly demonstrate that the pure SH-PEA is not cytotoxic at least up to the highest concentration evaluated (500 μg mL^−1^). The percentage of viable cells is always higher than 80%. The Janus Ag-PEG nanoparticles tested in different concentrations in the range of 4–250 μg mL^−1^ shows a high percentage (around 90%) of survival cells as its concentration is below 64 μg mL^−1^. Subsequently, a significant drop happens in their number for higher concentrations, and the drop of MCF-7 does faster. Instead, the multi-core shell Ag-PEG nanoparticles showed very high toxicity to HepG2 and MCF-7 cells. The percentage of viable cells for the multi-core shell nanoparticles against HepG2 is lower than 10% when its concentration is excess over 8 μg mL^−1^. To MCF-7, the number is only 81% even though the concentration of the nanoparticles is as low as 4 μg mL^−1^. The result demonstrates that the Janus Ag-PEG nanoparticles are more biocompatible than the multi-core shell with the investigated cells.

## 4. Conclusions

In summary, we synthesized Ag-PEG hybrid nanoparticles by photochemical reduction method with the as-synthesized SH-PEA as the coating, reducing and stabilizing agent. The condition of 3 min irradiation and 1 mg mL^−1^ SH-PEA is optimized for synthesizing stable Ag-PEG nanoparticles. The AgNO_3_ concentration plays a key role in tuning the morphologies of Ag-PEG nanoparticles. Janus Ag-PEG nanoparticles were synthesized with lower AgNO_3_ concentration, and multi-core shell structure with higher AgNO_3_ concentration. The pure AgNPs show spherical shape and have the size about 15 nm in the two structural Ag-PEG nanoparticles. Both structural Ag-PEG nanoparticles present strong antibacterial activities against *E. coli*, *S. aureus* and *B. subtilis*. The MIC and MBC values of the Janus nanoparticles are larger than those of the multi-core shell ones. The MBC/MIC ratio of the former is higher than 4, while the latter is lower than 4. HepG2 and MCF-7 cells were used to evaluate the cytotoxicity of the Ag-PEG nanoparticles. The Janus Ag-PEG nanoparticles show good biocompatibility when its concentration is below 64 μg mL^−1^, while the multi-core shell nanoparticles perform high toxicity even its concentration is as low as 8 μg mL^−1^. The results demonstrate the morphology of Ag-PEG hybrid nanoparticle has important effect on its biological properties.

## Figures and Tables

**Table 1:** The minimal inhibitory concentration (MIC) and minimum bacterial concentration (MBC) of Ag-PEG nanoparticles against *S. aureus*, *E. coli* and *B. subtilis*.

Bacterial Strain	Janus Structure (μg mL^−1^)			Multi-Core Shell Structure (μg mL^−1^)		
	MIC	MBC	MBC/MIC Ratio	MIC	MBC	MBC/MIC Ratio
*S. aureus*	64	500	8	16	64	4
*E. coli*	32	500	16	16	32	2
*B. subtilis*	64	250	4	16	32	2
